# Recurrent *NF1* gene variants and their genotype/phenotype correlations in patients with Neurofibromatosis type I

**DOI:** 10.1002/gcc.22997

**Published:** 2021-09-03

**Authors:** Matteo Riva, Davide Martorana, Vera Uliana, Edoardo Caleffi, Elena Boschi, Livia Garavelli, Giovanni Ponti, Luca Sangiorgi, Claudio Graziano, Stefania Bigoni, Luca Maria Rocchetti, Simona Madeo, Fiorenza Soli, Enrico Grosso, Diana Carli, Matteo Goldoni, Francesco Pisani, Antonio Percesepe

**Affiliations:** ^1^ Medical Genetics, Department of Medicine and Surgery University of Parma Parma Italy; ^2^ University Hospital of Parma Parma Italy; ^3^ Plastic Surgery, University Hospital of Parma Parma Italy; ^4^ Medical Genetics Unit, Azienda USL‐IRCCS Reggio Emilia Italy; ^5^ Division of Clinical Pathology University of Modena and Reggio Emilia Modena Italy; ^6^ Medical Genetics and Skeletal Rare Diseases, Istituto Ortopedico Rizzoli Bologna Italy; ^7^ Medical Genetics, S. Orsola‐Malpighi University Hospital Bologna Italy; ^8^ Medical Genetics, Ferrara University Hospital Ferrara Italy; ^9^ Medical Genetics, Maurizio Bufalini Hospital Cesena Italy; ^10^ Pediatrics, University Hospital of Modena Italy; ^11^ Medical Genetics, Santa Chiara Hospital Trento Italy; ^12^ Medical Genetics, Città della Salute e della Scienza University Hospital Torino Italy; ^13^ Department of Public Health and Pediatric Sciences University of Torino Torino Italy; ^14^ Statistics, Department of Medicine and Surgery University of Parma Parma Italy; ^15^ Children's Neuropsycological Services, Department of Medicine and Surgery University of Parma Parma Italy

**Keywords:** genotype/phenotype, Neurofibromatosis type I, *NF1* gene pathogenic variants

## Abstract

Neurofibromatosis type I, a genetic condition due to pathogenic variants in the *NF1* gene, is burdened by a high rate of complications, including neoplasms, which increase morbidity and mortality for the disease. We retrospectively re‐evaluated the *NF1* gene variants found in the period 2000–2019 and we studied for genotype/phenotype correlations of disease complications and neoplasms 34 variants, which were shared by at least two unrelated families (range 2–11) for a total 141 of probands and 21 relatives affected by Neurofibromatosis type I. Recurrent variants could be ascribed to the most common mutational mechanisms (C to T transition, microsatellite slippage, non‐homologous recombination). In genotype/phenotype correlations, the variants p.Arg440*, p.Tyr489Cys, and p.Arg1947*, together with the gross gene deletions, displayed the highest rates of complications. When considering neoplasms, carriers of variants falling in the extradomain region at the 5′ end of *NF1* had a lower age‐related cancer frequency than the rest of the gene sequence, showing a borderline significance (*p* = 0.045), which was not conserved after correction with covariates. We conclude that (1) hotspots in *NF1* occur via different mutational mechanisms, (2) several variants are associated with high rates of complications and cancers, and (3) there is an initial evidence toward a lower cancer risk for carriers of variants in the 5′ end of the *NF1* gene although not significant at the multivariate analysis.

## 1 INTRODUCTION

Neurofibromatosis type I (MIM#162200), a genetic condition featured by various combinations of multiple café‐au‐lait spots, axillary and inguinal freckling, multiple cutaneous and subcutaneous neurofibromas, and iris Lisch nodules, is burdened by a high rate of complications, such as optic pathway gliomas, plexiform neurofibromas, osseous dysplasias, and cancers, which can severely impair the quality of life of the affected patients and finally increase the morbidity and mortality for the disease.^1^, ^2^ In Neurofibromatosis type I, malignancies reduce the average life expectancy of the affected patients by 10–15 years and represent the main cause of death for the disease.^2^

The responsible gene, *Neurofibromin 1* (*NF1*, MIM *613113), an oncosuppressor acting as a negative regulator of the Rat sarcoma (Ras) cascade, is characterized by a high rate of de novo pathogenic variants (about 50% of the affected patients harbor novel alleles) and by the substantial absence of hot spots, which have built up our knowledge of the disease as a combination of individual clinical histories with a high inter‐ and intra‐familial phenotypic variability.^3^, ^4^ Our understanding of the genotype/phenotype correlations for Neurofibromatosis type I has also been hampered by technical and clinical issues, which have reduced the number of patients undergoing to the genetic test for the disease.^5^, ^6^ In fact, both the large size of the gene, composed of 61 coding exons, and a selective indication to perform the genetic testing have brought to a little use of the genetic information, which is not needed to confirm often obvious phenotypes and is not able to predict the clinical outcome for most of the *NF1* genetic variants, with few exceptions, such as large deletions having a severe phenotype and other missense and inframe deletions (p.Arg1809, p.Met1149, and p.Met992del) associated with the absence of cutaneous neurofibromas.^7^, ^8^, ^9^, ^10^, ^11^, ^12^ All these reasons have confined the molecular analysis to a minority of the affected patients and to the diagnostic dilemmas, mostly children with a discrete number of café‐au‐lait (CAL) spots with no other associated feature, with few possibilities to perform correlations for degenerative disorders, like cancer, related to the type and site of *NF1* variant.^13^ In recent years, instead, the advent of the next‐generation sequencing (NGS) and the raising demand for pre‐implantation diagnosis have increased the access to the genetic test, which is revealing new aspects of the disease, like the features of the novel variants,^14^ the presence of selected hotspots (i.e., p.Met992del, p.Met1149, p.Arg1809, p.Arg1276, and p.Lys1423), and the genotype/phenotype correlations for specific codons, like the 844–848.^7^, ^8^, ^9^, ^15^, ^16^, ^17^, ^18^ A positive genetic testing with the finding of a heterozygous pathogenic *NF1* variant has been recently incorporated in the revised International Consensus Criteria for the diagnosis of Neurofibromatosis type I.^19^

The issue of genotype/phenotype correlations in Neurofibromatosis type I is further complicated by the mechanism of action of the *NF1* gene, which acts as an oncosuppressor through the combination of a constitutional variant (inherited or de novo) and a somatic, acquired inactivation of the other allele for the initiation of the molecular cascade in most of the affected tissues.^20^, ^21^ The randomness and variety of the second hit (loss of heterozygosity, point mutations, epigenetic inactivations) have for long time made the phenotype unpredictable based on the constitutional variant alone, making the genotype/phenotype correlations difficult.^22^, ^23^

To reduce the impact of the constitutional variability and to address the issue of the recurrence of variants in the *NF1* gene, in the present study we analyzed the genotype/phenotype correlations of patients bearing the same constitutional pathogenic variant, by selecting from our large record of 1007 patients 34 *NF1* recurrent variants (those carried by at least two unrelated patients) and by correlating them with the Neurofibromatosis type I phenotype, in particular with the onset of complications and cancers.

## 2 METHODS

### 2.1 Patients

The analysis is based on the records of Parma University Hospital's Unit of Medical Genetics, covering the period January 2000 to December 2019. The laboratory functions as hub for the entire Emilia Romagna Region (4.5 million inhabitants) and attracts patients also from other Italian regions. Genetic testing was performed on patients having a clinical suspicion of Neurofibromatosis type I based on the presence of at least two of the clinical manifestations proposed by the National Institute of Health Consensus Development Conference, that is, the presence of six or more CALs > 15 mm in adults and > 5 mm in children, two or more cutaneous/subcutaneous neurofibromas or one or more plexiform neurofibromas, axillary/inguinal freckling, two or more Lisch nodules, optic pathway gliomas, a distinctive osseous lesion such as sphenoidal dysplasia or long‐bone dysplasia, and a first‐degree relative with Neurofibromatosis type 1 diagnosed by the above criteria (NIH Consensus Development Conference, 1988). Phenotypes of segmentary Neurofibromatosis type I were not included in the study. Each sample sent to our Laboratory was accompanied by a chart in which a clinical description of the patient by the referring physician was reported, with special reference to the presence/absence of the typical phenotypic features of the disease and their age of diagnosis. Patient's clinical records, genealogical trees and genetic test results were all collected, anonymized, and archived in a dedicated Excel file in which the clinical data are updated to the last follow‐up control. In the period 2000–2019, 1077 subjects with a clinical suspicion of Neurofibromatosis type I were subjected to genetic testing as part of their diagnostic process.

### 2.2 NF1 genetic test

*NF1* genetic test has been previously described.^14^ Briefly, from April 2000 to June 2016, genetic analyses on genomic DNA were conducted using denaturing high pressure liquid chromatography (DHPLC) and sequencing of samples with a profile difference from a wild‐type control, whereas, starting from June 2016, the laboratory has switched to NGS using the Illumina MiSeq platform (TruSeq Custom Amplicon v.1.5) according to standard protocols.^14^ Starting from 2005, the negative samples at the sequencing analysis have been subjected to Multiplex Ligation‐dependent Probe Amplification (MLPA, MRC Holland) for the detection of deletions or duplications spanning one or more exons. The combined approach of NGS and MLPA has a reported detection rate of 88%,^23^ higher than the 72% of the previously used DHPLC technique,^24^ reflecting the technical changes over the years but not substantially affecting the results of the study, which is based on the mutation‐positive cases.

The description and nomenclature of sequence variations at DNA and protein level have been done according to the Mutalyzer^25^ software version 2.0.32 with the NM_000267.3 reference sequence. Classification of the genetic variants has been performed through the classical 5‐tiered system, based on standard criteria.^26^

### 2.3 The database

The recurrent *NF1* gene variants were extracted from the diagnostic records and included in an anonymized file together with the genetic information (type, effect, amino acid change, exon involved, protein domain, segregation ‐de novo/familial‐). The studied gene variants have been submitted to ClinVar^27^ (Accession numbers: SCV001218909 to SCV001218931) and Leiden open variant database (LOVD).^28^ In addition, the main clinical features of the patients with recurrent pathogenic variants were recorded according to the human phenotype ontology^29^ (HPO), after reanalyzing the original documents and, when incomplete, by consulting the referring physician. All the features requiring specific follow‐up and/or therapy were recorded as complications, as follows: cognitive impairment, epilepsy, short stature, symptomatic glioma (including optic nerve and other central nervous system), scoliosis (requiring orthesis or surgical treatment), sphenoidal or long bone dysplasia, spinal neurofibroma, severe plexiform neurofibroma (> 3 cm), cardiovascular complications, including congenital septal and valvular defects, cardiomyopathies, and hypertension, both essential or nephrovascular. The age of onset of complication is recorded both prospectively (when the complication is diagnosed during follow‐up) and retrospectively, through the analysis of previous clinical records of the patient. In the presence of multiple complications, the age of onset refers to the earliest manifestation. Neoplastic complications were recorded according to the site of onset of the primary tumor.

The statistical analysis has been carried out with the R^30^ and with the IBM SPSS Statistics for Windows, Version 22 (IBM Corp., Armonk, NY) software, using the chi‐squared test for comparisons among groups and the log‐rank test for ascertaining differences in age‐related frequency of complications or neoplasms. When multiple tests were performed, the significance of the *p*‐values was adjusted according to the Benjamini–Hochberg procedure. Logistic regression analysis to evaluate the independent contribution of the clinical and genetic features to cancer onset was performed according to the Cox's model. The study has been approved by the local ethical committee (Prot. N. 29627/2019).

## 3 RESULTS

### 3.1 Recurrent variants

**FIGURE 1:**
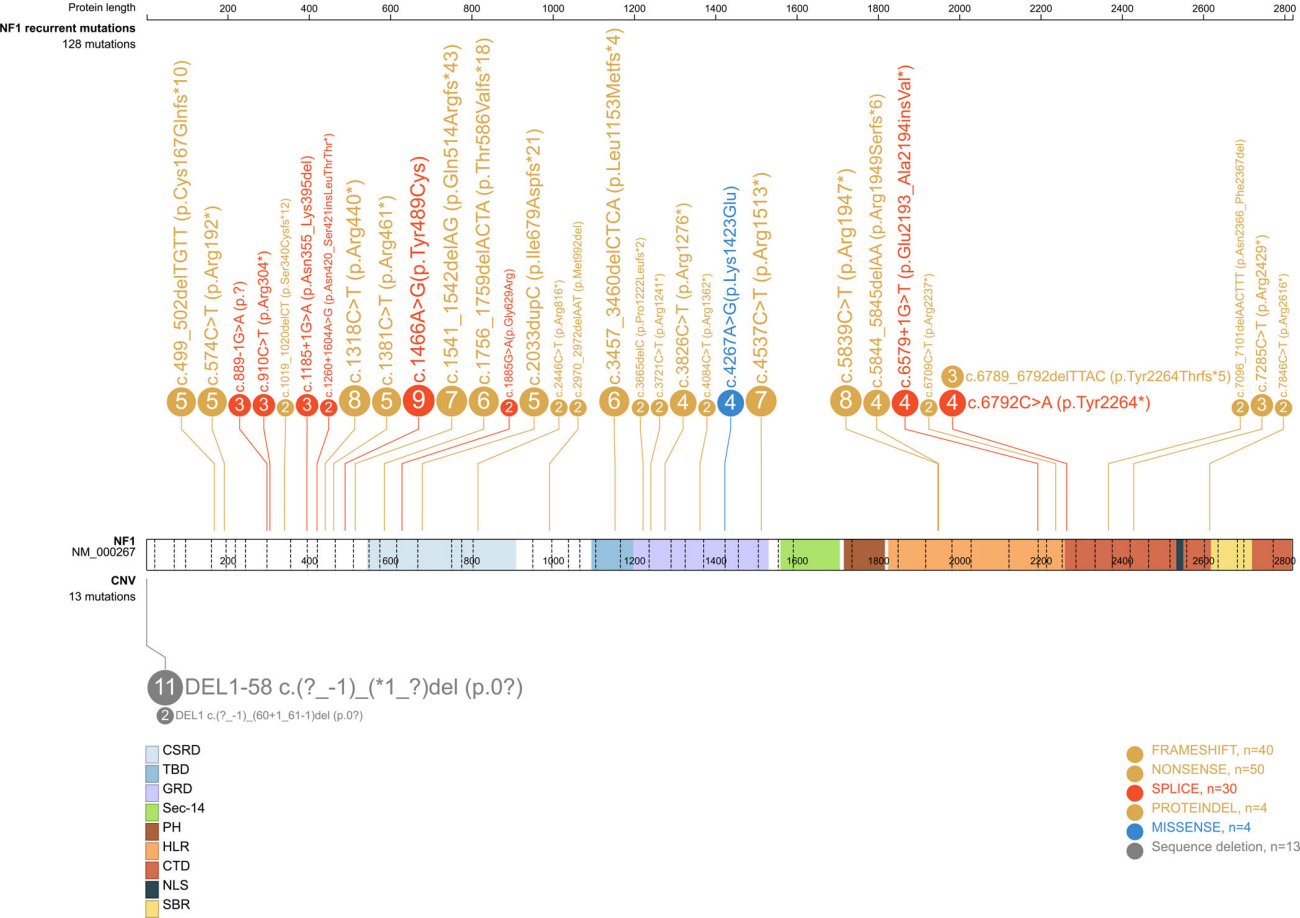
Diagram representing the *NF1* gene with colors varying according to the protein domains (in white the extradomain regions). The numbers into the circles refer to the probands sharing the same constitutional variant; the color of the circle is specific for the type of variant (copy number variant [CNV], truncating, splice site, missense)

The analysis of our records including 1077 gene variants revealed 141 unrelated NF1 affected probands who shared 34 variants, each of them recurring at least 2 times in apparently unrelated families. Table 1 shows their frequency distribution, ranging from 2 (12 variants, 24 probands) to 11 (whole gene deletions, shared by 11 probands), their type, and the demographic features of the patients. All the variants are listed in Table 2 and their distribution along the gene is shown in Figure 1: of note, 12 variants out of 34 reside in the 5′ extradomain region (35%, 95% confidence interval [CI] 21.5–52.1), 13 out of the 17 single‐base changes (nonsense and missense) are C>T transitions (76%, 95% CI 52.7–90.4), 3 out the 11 indels are 2‐,4‐,6‐basepair microsatellite deletions (27%, 95% CI 9.7–56.6).

**TABLE 1:** Descriptive table of the number and type of recurring NF1 variants and demographic characteristics of the carrier patients

	Frequency of recurrence									
	2	3	4	5	6	7	8	9	11	Total
Number of variants	12	5	5	4	2	2	1	2	1	34
Type of variant										
Gross deletion	1	0	0	0	0	0	0	0	1	2
Indels	4	1	1	2	2	1	0	0	0	11
Splice site	2	3	2	0	0	0	0	1	0	8
Nonsense	5	1	1	2	0	1	1	1	0	12
Missense	0	0	1	0	0	0	0	0	0	1
Familiar/sporadic (probands and family members)										
Familiar	2	7	7	4	0	4	0	13	0	37
Sporadic	23	12	17	18	12	12	8	12	11	125
Family members (excluding probands)	1	4	4	2	0	2	0	8	0	21
Sex (total no. of probands and family members)										
Male	7	6	12	9	4	6	5	9	7	65
Female	18	13	12	13	8	10	3	16	4	97
Age (total no. of probands and family members)										
<18 years	10	8	9	9	6	6	2	13	6	69
≥18 years	15	11	15	13	6	10	6	12	5	93

**TABLE 2:** List of the recurrent mutations and the phenotypic features referred to as complications and cancers

	Probands	Family members	Total points	Complications*	Cancers
	*n*	*n*	*n*	*n* (%)	*n* (%)
c.(?_‐1)_(*1_?)del (p.0?)	11	‐	11	7	‐
<18	6	‐	6	4 (66.7)	‐
≥18	5	‐	5	3 (60)	‐
c.(?_‐1)_(60+1_61‐1)del (p.0?)	2	‐	2	2	‐
<18	1	‐	1	1 (100)	‐
≥18	1	‐	1	1 (100)	‐
c.499_502delTGTT(p.Cys167Glnfs*10)	5	2	7	4	1
<18	3	‐	3	2 (66.7)	‐
≥18	2	2	4	2 (50)	1 (25)
c.574C>T(p.Arg192*)	5	‐	5	1	1
<18	3	‐	3	‐	‐
≥18	2	‐	2	1 (50)	1 (50)
c.889‐1G>A (p.?)	3	‐	3	2	‐
<18	1	‐	1	‐	‐
≥18	2	‐	2	2 (100)	‐
c.910C>T(p.Arg304*)	3	1	4	2	‐
<18	1	1	2	1 (50)	‐
≥18	2	‐	2	1 (50)	‐
c.1019_1020delCT(p.Ser340Cysfs*12)	2	‐	2	1	1
<18	1	‐	1	‐	‐
≥18	1	‐	1	1 (100)	1 (100)
c.1185+1G>A (p.Asn355_Lys395del)	3	‐	3	‐	‐
<18	2	‐	2	‐	‐
≥18	1	‐	1	‐	‐
c.1260+1604A>G (p.Asn420_Ser421insLeuThrThr*)	2	‐	2	1	1
<18	‐	‐	‐	‐	‐
≥18	2	‐	2	1 (50)	1 (50)
c.1318C>T (p.Arg440*)	8	‐	8	6	1
<18	2	‐	2	2 (100)	‐
≥18	6	‐	6	4 (66.7)	1 (16.7)
c.1381C>T (p.Arg461*)	5	‐	5	2	1
<18	2	‐	2	1 (50)	‐
≥18	3	‐	3	1 (33.3)	1 (33.3)
c.1466A>G (p.Tyr489Cys)	9	2	11	7	‐
<18	4	2	6	3 (50)	‐
≥18	5	‐	5	4 (80)	‐
c.1541_1542delAG (p.Gln514Argfs*43)	7	1	8	4	‐
<18	3	‐	3	1 (33.3)	‐
≥18	4	1	5	3 (60)	‐
c.1756_1759delACTA (p.Thr586Valfs*18)	6	‐	6	3	1
<18	3	‐	3	2 (66.7)	‐
≥18	3	‐	3	1 (33.3)	1 (33.3)
c.1885G>A (p.Gly629Arg)	2	‐	2	‐	1
<18	1	‐	1	‐	‐
≥18	1	‐	1	‐	1 (100)
c.2033dupC (p.Ile679Aspfs*21)	5	‐	5	3	‐
<18	1	‐	1	‐	‐
≥18	4	‐	4	3 (75)	‐
c.2446C>T(p.Arg816*)	2	‐	2	‐	1
<18	1	‐	1	‐	‐
≥18	1	‐	1	‐	1 (100)
c.2970_2972delAAT (p.Met992del)	2	‐	2	‐	1
<18	1	‐	1	‐	‐
≥18	1	‐	1	‐	1 (100)
c.3457_3460delCTCA (p.Leu1153Metfs*4)	6	‐	6	4	3
<18	3	‐	3	2 (66.7)	1 (33.3)
≥18	3	‐	3	2 (66.7)	2 (66.7)
c.3665delC (p.Pro1222Leufs*2)	2	‐	2	‐	1
<18	2	‐	2	‐	1 (50)
≥18	‐	‐	‐	‐	‐
c.3721C>T (p.Arg1241*)	2	‐	2	2	‐
<18	1	‐	1	1 (100)	‐
≥18	1	‐	1	1 (100)	‐
c.3826C>T (p.Arg1276*)	4	2	6	3	2
<18	2	‐	2	1 (50)	‐
≥18	2	2	4	2 (50)	2 (50)
c.4084C>T (p.Arg1362*)	2	‐	2	2	‐
<18	‐	‐	‐	‐	‐
≥18	2	‐	2	2 (100)	‐
c.4267A>G (p.Lys1423Glu)	4	‐	4	1	‐
<18	3	‐	3	1 (33.3)	‐
≥18	1	‐	1	‐	‐
c.4537C>T (p.Arg1513*)	7	1	8	6	1
<18	3	‐	3	2 (66.7)	‐
≥18	4	1	5	4 (80)	1 (20)
c.5839C>T (p.Arg1947*)	8	6	14	6	2
<18	3	4	7	2 (25)	1 (12.5)
≥18	5	2	7	4 (57.1)	1 (14.3)
c.5844_5845delAA (p.Arg1949Serfs*6)	4	‐	4	2	3
<18	‐	‐	‐	‐	‐
≥18	4	‐	4	2 (50)	3 (75)
c.6579+1G>T (p.Glu2193_Ala2194insVal*)	4	2	6	2	‐
<18	2	1	3	1 (33.3)	‐
≥18	2	1	3	1 (33.3)	‐
c.6709C>T (p.Arg2237*)	2	‐	2	1	‐
<18	‐	‐	‐	‐	‐
≥18	2	‐	2	1 (50)	‐
c.6789_6792delTTAC (p.Tyr2264Thrfs*5)	3	1	4	2	1
<18	‐	‐	‐	‐	‐
≥18	3	1	4	2 (50)	1 (25)
c.6792C>A(p.Tyr2264*)	4	‐	4	1	1
<18	1	‐	1	‐	‐
≥18	3	‐	3	1 (33.3)	1 (33.3)
c.7096_7101delAACTTT(p.Asn2366_Phe2367del)	2	‐	2	‐	1
<18	1	‐	1	‐	‐
≥18	1	‐	1	‐	1 (100)
c.7285C>Tp.(Arg2429*)	3	2	5	2	0
<18	2	1	3	2 (66.7)	‐
≥18	1	1	2	‐	‐
c.7846C>Tp.(Arg2616*)	2	1	3	‐	1
<18	‐	1	1	‐	‐
≥18	2	‐	2	‐	1 (50)
Total	141	21	162	79	26

*Note*: Patients are divided into two age categories (<18 and ≥18).

### 3.2 *NF1* gene variants and disease complications

**FIGURE 2:**
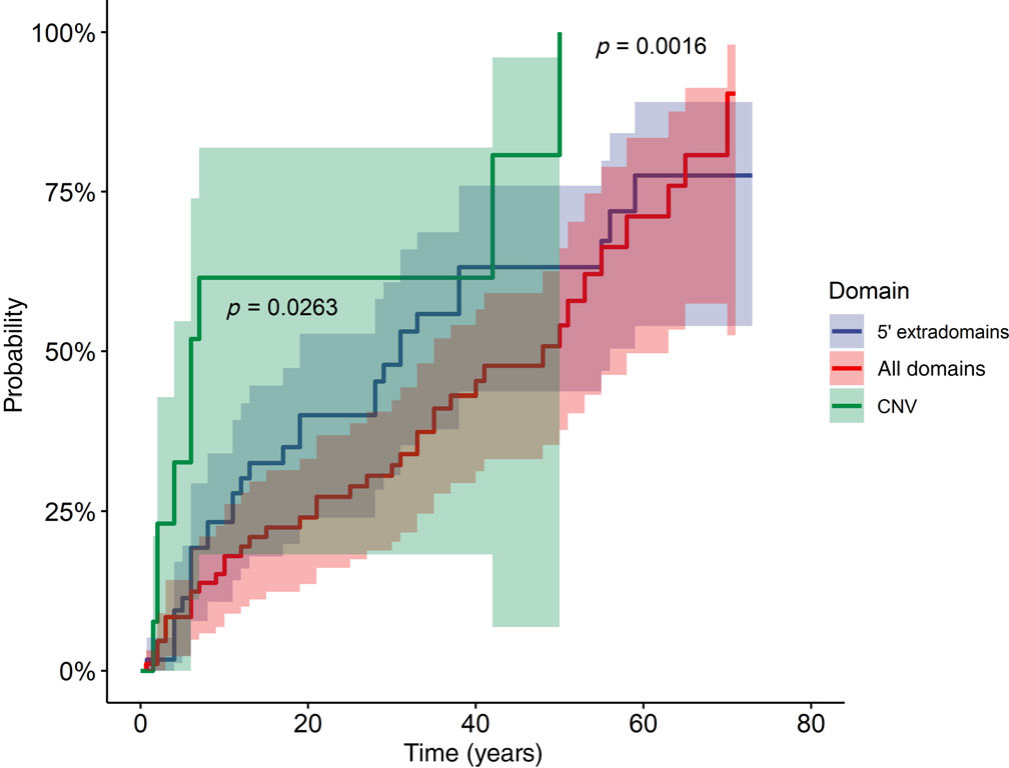
Penetrance curves showing the cumulative probability of developing complications (any site) for patients with *NF1* variants divided according to the variant region (copy number variants [CNVs], 5′ extradomain region vs. all the other domains of the gene). *p*‐values, set at < 0.034 after adjustment for multiple test according to the Benjamini–Hochberg procedure, refer to the log‐rank results using CNVs as reference category versus those in the 5′ extradomain region (blue line, *p* = 0.026) and versus all the other domains of the gene (red line, *p* = 0.002). The shaded areas indicate the 95% confidence intervals

A genotype/phenotype analysis was performed for the 141 probands under study and their 21 family members for investigating the specific rates of complications for each of the *NF1* gene recurrent variants, dividing the patients into two age categories (< 18 and ≥ 18 years) (Tables 2 and S1). Besides the high frequency of complications for the young patients carrying whole *NF1* gene deletions (4 out of 6, 67%, 95% CI 30–90.3), there were other variants with a high frequency of disease complications: 67% of the adult cases (4 out of 6, 95% CI 30–90.3) and both the young patients with the c.1318C>T (p.Arg440*), 80% (95% CI 37.5–96.4) (4 out of 5) of the adults and 50% (3 out of 6, 95% CI 18.8–81.2) of the young with the c.1466A>G (p.Tyr489Cys) and 57% (95% CI 25.0–84.2) (4 out of 7) of the adult patients with the c.5839C>T (p.Arg1947*) presented complications related to the Neurofibromatosis type I (Tables 2 and S1). Two variants instead, shared by seven patients, were not associated with any complication: the c.1185+1G>A (three unrelated patients of 12, 15, and 27 years of age) and the c.4267A>G (p.Lys1423Glu) (four unrelated patients of 1, 2, 3, and 37 years of age). By grouping the recurrent variants according to their localization in the *NF1* sequence (for this purpose, the NF1 protein domains were used, see Figure 1), no significant differences (*χ* ^2^ 9.5, *p* = 0.2) emerged among the groups for the overall rate of complications (Table 3). When the age‐dependent penetrance of complications was analyzed, the known higher frequency of complications for the copy number variants (CNVs) of the *NF1* gene was confirmed, when compared with the variants in the extradomain region at the 5′ end or to the rest of the gene sequence (log‐rank = 4.93, *p* = 0.026 and log‐rank = 9.97, *p* = 0.0016, respectively, Figure 2).

**TABLE 3:** Number and percentage of patients with complications and neoplasm, grouped by the protein domain of the NF1 gene (gross deletions represent a separate group)

	Gross deletions	5′ extradomain	CSRD domain	Intagenic extradomain	TBD domain	GRD domain	HLR domain	CTD domain	*p*‐value
No. of patients	13	58	15	2	6	24	26	18	
No. of variants	2	11	4	1	1	6	4	5	
Median age (range)	13 (1–50)	25 (6 mo‐73)	33 (1–65)	19 (3–35)	32.5 (2–69)	18.5 (8 mo‐65)	21 (1–70)	31.5 (1–71)	
Complications (%)									
Scoliosis (HP:0002650)	1 (7.7)	2 (3.4)	‐	‐	1 (16.7)	‐	‐	‐	
Bone dysplasia (HP:0010734)	‐	4 (6.9)	‐	‐	1 (16.7)	1 (4.2)	‐	‐	
Cognitive impairment (HP:0100543)	4 (30.8)	11 (19)	2 (13.3)	‐	1 (16.7)	5 (20.8)	4 (14.8)	2 (11.1)	
Short stature (HP:0004322)	1 (7.7)	‐	‐	‐	‐	1 (4.2)	‐	‐	
Symptomatic glioma^a^ (HP:0009734)	‐	3 (5.2)	1 (6.7)	‐	‐	1 (4.2)	‐	‐	
Cardiovascular^b^	2 (15.4)	3 (5.2)	‐	‐	‐	4 (16.7)	2 (7.4)	‐	
Spinal neurofibroma (HP:0009735)	‐	1 (1.7)	1 (6.7)	‐	‐	‐	2 (7.4)	‐	
Plexiform neurofibroma (HP:0009732)	1 (7.7)	5 (8.6)	2 (13.3)	‐	1 (16.7)	2 (8.3)	3 (11.1)	3 (16.7)	
Epilepsy (HP:0001250)	‐	1 (1.7)	‐	‐	‐	‐	‐	‐	
Total (%; 95% CI)	9 (69.2; 42.4–87.3)	30 (53.4; 39.2–64.1)	6 (40; 19.8–64.2)	‐	4 (66.8; 30–90.3)	14 (62.6; 42.7–78.9)	11 (40.7; 24.5–59.3)	**5(27.8; 12.5–50.9)**	**0.201**
Cancer (%)									
GIST (HP:0100723)	‐	2 (3.4)	‐	‐	‐	1 (4.2)	‐	‐	
Breast cancer (HP:0003002)	‐	2 (3.4)	‐	1 (50)	‐	‐	‐	1 (5.6)	
MPNST (HP:0100697)	‐	1 (1.7)	‐	‐	‐	‐	‐	‐	
Astrocytoma (HP:0009592)	‐	‐	‐	‐	‐	1 (4.2)	2 (7.4)	‐	
Pheochromocytoma (HP:0002666)	‐	‐	‐	‐	1 (16.7)	‐	‐	‐	
Meningioma (HP:0002858)	‐	‐	1 (6.7)	‐	‐	‐	1 (3.7)	‐	
Colon cancer (HP:0003003)	‐	1 (1.7)	‐	‐	‐	‐	‐	‐	
Duodenal cancer (HP:0006771)	‐	‐	1 (6.7)	‐	‐	‐	‐	‐	
Juvenile myelomonocytic leukemia (HP:0012209)	‐	‐	‐	‐	‐	1 (4.2)	‐	‐	
Malignant glioma (HP:0009733)	‐	‐	‐	‐	‐	1 (4.2)	‐	‐	
Multiple myeloma (HP:0006775)	‐	‐	‐	‐	‐	‐	‐	1 (5.6)	
Myxofibrosarcoma (HP:0030448)	‐	‐	‐	‐	‐	‐	‐	1 (5.6)	
Neuroblastoma (HP:0003006)	‐	‐	‐	‐	1 (16.7)	‐	‐	‐	
Lung cancer (HP:0100526)	‐	‐	‐	‐	‐	‐	1 (3.7)	‐	
Rhabdomyosarcoma (HP:0002859)	‐	‐	‐	‐	‐	‐	1 (3.7)	‐	
Rectal adenocarcinoma (HP:0100743)	‐	‐	‐	‐	1 (16.7)	‐	‐	‐	
Sarcoma (HP:0100242)	‐	‐	‐	‐	‐	‐	‐	1 (5.6)	
Vater's ampulla carcinoid (HP:0006722)	‐	‐	1 (6.7)	‐	‐	‐	‐	‐	
Total (%; 95% CI)	‐	6 (10.3; 4.8–20.8)	3 (20; 7.0–45.2)	1 (50; 9.4–90.5)	3 (50; 18.8–81.2)	4 (16.7; 6.7–35.9)	5 (18.5; 8.2–36.7)	**4 (22.2; 9.0–45.2)**	**0.083**

*Note*: For patients with multiple complications and/or cancers, the table reports only the most clinically relevant manifestation and the earliest neoplasm, respectively.

^a^ Include optic nerve glioma (HP:0009734) and other CNS glioma (HP:0009733).

^b^ Include patency of foramen ovale (HP:0001655), diastolic dysfunction (HP:0005117), venous malformation (HP:0012721) of brain, aneurysm (HP:0002617) of vertebral artery, cardiac malformation (HP:0001627), cardiac fibroma (HP:0010617), hypertrophic cardiomyopathy (HP:0001639), pulmonic valve stenosis (HP:0001642), mitral valve prolapse (HP:0001634), pulmonary stenosis (HP:0004415), and essential hypertension (HP:0000822).

### 3.3 *NF1* gene variants and cancer

As far as the neoplastic complications of the disease are concerned, a high frequency of cancer complications was observed for two variants: the c.5844_5845delAA (p.Arg1949Serfs*6) with three cases of cancer out of four unrelated adult patients and the c.3457_3460delCTCA (p.Leu1153Metfs*4) with three cases of cancer in six unrelated patients (Table 2). Multiple tumors were present in five unrelated patients, one of whom had a concurrent diagnosis of Lynch syndrome and showed breast, endometrial, and colon cancers (the two latter neoplasms, typical of the Lynch syndrome, were excluded from further analysis).

**FIGURE 3:**
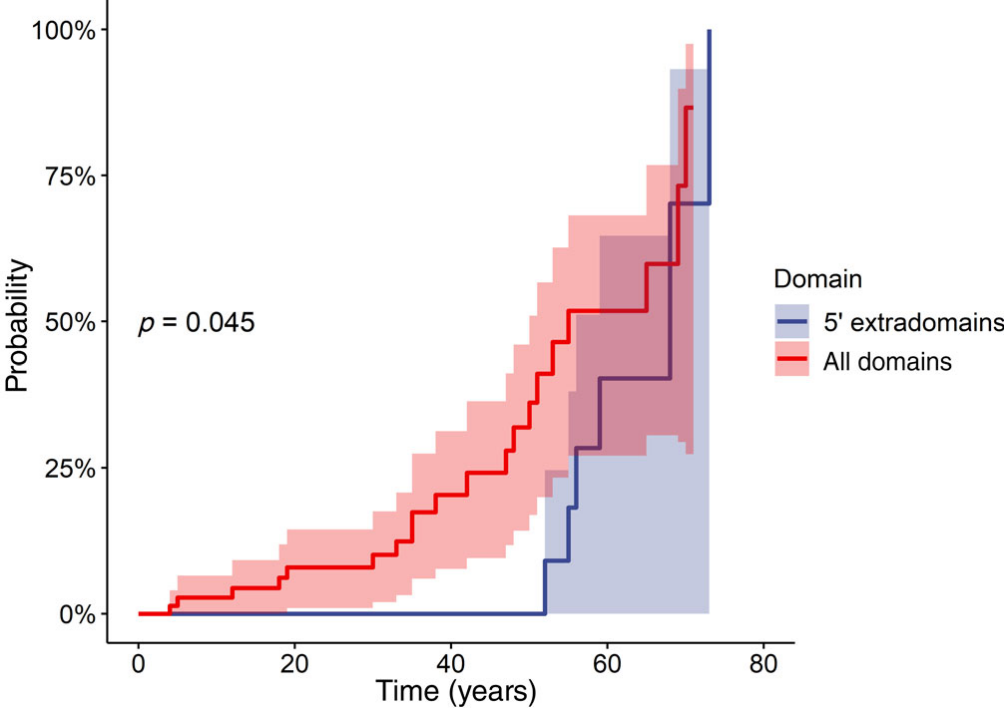
Penetrance curves showing the cumulative probability of developing cancer (any site) for patients with *NF1* variants divided according to the mutation region (5′ extradomain region vs. all the other domains of the gene). Coy number variants are not reported, due to the absence of incident cases in the study population. The shaded areas indicate the 95% confidence intervals

No significant differences in the frequency of cancer occurrence were found after grouping the recurrent variants according to their localization into domains (*χ* ^2^ 11.47, *p* = 0.08), but a significantly lower age‐related cancer frequency was observed for the variants located in the extradomain region at the 5′ end, compared to the rest of the gene sequence (log‐rank = 4.00, *p* = 0.045) (Figure 3). However, correction with covariates (age, sex) in Cox's regression yielded no significant result.

## 4 DISCUSSION

Neurofibromatosis type I is a long‐standing model of clinical and genetic variability, which is due to the pleiotropic expression and to the mutational pattern of the *NF1* gene^13^, ^15^ and is characterized by the frequent occurrence of de novo variants and their dispersion through the whole gene, with only few preferential locations or mechanisms.^1^, ^4^ The present study, which analyses the recurrence of the gene variants, confirms that there is not a unique mutational hotspot in the *NF1* gene, although some variants occur with higher frequency, like the c.1466A>G,^31^ which we have found in nine unrelated families and is otherwise one of the most common variant in the LOVD^28^ (43 reports by August 2021) and ClinVar (16 reports) databases. With reference to the origin of the *NF1* recurrent variants, the full penetrance of Neurofibromatosis I argues against the hypothesis of the segregation of a founder mutation, common to several diseases in humans^32^ and points to the presence of universal mutational mechanisms. In fact, 13 out of the 34 recurrences in our study are C to T transitions, which are among the most frequent causes of human genetic disease phenotypes and account for a substantial fraction of the polymorphic variability of the human genome through the methylation–deamination of cytosines.^33^ Moreover, the universality of this mutational mechanism is indicated by the finding that 8 of our recurrent nonsense variants (including codons 192, 304, 440, 816, 1241, 1362, 1513, and 2429) have been previously reported not only as germline but also as somatic variations.^34^, ^35^ On the other hand, when analyzing the recurrent variants with an underlying insertion/deletion mechanism, 3 out of 11 are featured by gains or losses of nucleotides from microsatellite tracts (of 2‐, 4‐, and 6‐bp repeats) through another ubiquitary mutational mechanism subtended by the misalignment and slippage during the DNA replication.^36^ Finally, also the other large group of recurrences, that is, the *NF1* gene rearrangements (whole gene and exon 1 deletions for a total of 13 families), can be ascribed to one of the most common mechanisms of variability of the human genome, which is mediated by the homologous non allelic recombination during meiosis causing a DNA CNV.^37^ More in general, the whole mutational landscape of the Neurofibromatosis type I patients appears related to the gene context and length, differently from other examples of constitutional variability, like *FGFR3* or *FGFR2*, which show hotspots, a single mutational mechanism causing a gain‐of‐function and a clear correlation to the paternal age, consistent with the increased number of cell divisions occurring in aging spermatogonia.^38^ In *NF1*, a correlation with the paternal age has been proposed, but not confirmed by all the studies by our group and others,^14^, ^39^, ^40^, ^41^, ^42^ possibly due to the still low number of observations.

Concerning the specific genotype/phenotype correlation, apart from the whole‐gene deletions, which show the known high frequency of cognitive impairment (Figure 2),^16^ the rate of complications or neoplasms presents a wide range (Table 2), regardless of the type of variant (truncating or non‐truncating): for example, the p.Met992del in‐frame deletion, which is expected to generate a milder phenotype,^7^ resulted in our study in a breast cancer at 35 years of age. Although breast cancer is part of the neoplastic spectrum of the Neurofibromatosis I disease,^43^ the high genetic heterogeneity of this neoplasm does not rule out in the specific case the possible contribution of different genetic predisposition factors or the role of other modifier genes in the phenotypic expression of the disease.^44^ Also the data on the p.Arg1947*, which is reported with a milder phenotype,^45^ are only partly confirmed, since we have found 2 tumors (one rhabdomyosarcoma at 19 year of age and one hypothalamic astrocytoma at 12) out of the 14 patients harboring the variant. Finally, our data support the association with cardiovascular anomalies of the p.Lys1423Glu variant,^9^ which was found in one child with pulmonary valve stenosis (Table 2).

When the variants were grouped according to their *NF1* gene region, those located in the 5′ end preceding the first protein domain (Figure 1) showed a significantly lower age‐specific tumor burden compared to all the other domains together (Figure 3), consistent with the model of other oncosuppressors like the *APC* gene, which causes an attenuated polyposis in carriers of pathogenic variants at the 5′ end.^46^ A cautious interpretation of this finding is of course mandatory, due to the lack of significance for the role of that gene region on cancer onset at the multivariate analysis, to the possibility of a type 1 statistical error for multiple testing and to the obvious need of confirmation by larger, independent series, taking into account all the *NF1* gene mutation burden and not limiting the analysis to the recurrent variants only, like in the present study. As to the tumor spectrum, there was no specific association among variants and tumor sites and most of the diagnosed neoplasms belong to the typical spectrum of Neurofibromatosis type I, involving malignancies of the nervous system (central, peripheral, and autonomic), in particular MPNSTs, soft tissues tumors, hematopoietic cancers, pheochromocytoma, and breast cancers. Only in two cases, lung and rectal adenocarcinomas,^34^, ^47^, ^48^, ^49^ the tumor site is not typical, although the association of both neoplasms with Neurofibromatosis type I has been reported in specific case‐reports.^50^, ^51^

In conclusion, our study on recurrent *NF1* variants shows that the features of recurrence in the *NF1* gene are not univocal but involve the most common mutational mechanisms in humans; furthermore, the genotype/phenotype correlation indicates that several variants are associated with a very high risk of disease complications and suggests an initial link between variants in the 5′ end of the gene and a lower risk of cancer complications of the disease, although not significant at the multivariate analysis.

## AUTHOR CONTRIBUTIONS

Conceptualization: Davide Martorana, Vera Uliana and Antonio Percesepe. Data curation: Matteo Riva, Davide Martorana and Matteo Goldoni. Methodology: Matteo Goldoni. Resources: Edoardo Caleffi, Elena Boschi, Livia Garavelli, Giovanni Ponti, Luca Sangiorgi, Claudio Graziano, Stefania Bigoni, Luca Maria Rocchetti, Simona Madeo, Fiorenza Soli, Enrico Grosso and Francesco Pisani. Software: Diana Carli. Writing—original draft: Matteo Riva. Writing—review and editing: Francesco Pisani and Antonio Percesepe.

## Supporting information


**Table S1.** Summary of the complications and cancers diagnosed in the patients under study. Age at follow‐up refers to the last follow‐up visit

## Data Availability

Data are available on request from the authors.
