# Supplementary material for: Recurrent NF1 gene variants and their genotype/phenotype correlations in patients with Neurofibromatosis type I
Source: Genes Chromosomes Cancer. 2021 Sep 3;61(1):10–21. doi: 10.1002/gcc.22997 (PMC9291954; doi:10.1002/gcc.22997)
Supplement: Supplementary file 1 — Table S1. Summary of the complications and cancers diagnosed in the patients under study. Age at follow‐up refers to the last follow‐up visit [file GCC-61-10-s001.docx]

**SUPPLEMENTARY TABLE 1 - Summary of the complications and cancers diagnosed in the patients under study. Age at follow-up refers to the last follow-up visit**

| **CODE** | **VARIANT** | **AGE AT FOLLOW-UP** | **CANCER** | **COMPLICATIONS** |
| --- | --- | --- | --- | --- |
| PR001 | DEL1-58:c.(?_-1)_(*1_?)del (p.0?) | 50.0 |  | Dystrophic scoliosis |
| PR002 | DEL1-58:c.(?_-1)_(*1_?)del (p.0?) | 33.0 |  | Macrocephaly, plexiform neurofibroma, cognitive impairment |
| PR003 | DEL1-58:c.(?_-1)_(*1_?)del (p.0?) | 4.0 |  | Short stature |
| PR004 | DEL1-58:c.(?_-1)_(*1_?)del (p.0?) | 2.0 |  | Cardiovascular alterations (patency of foramen ovale and mild pulmonary stenosis) |
| PR005 | DEL1-58:c.(?_-1)_(*1_?)del (p.0?) | 42.0 |  | Plexiform neurofibroma |
| PR006 | DEL1-58:c.(?_-1)_(*1_?)del (p.0?) | 1.5 |  | Cardiovascular alterations (Pulmonic valve stenosis ) |
| PR007 | DEL1-58:c.(?_-1)_(*1_?)del (p.0?) | 13.0 |  | Cardiovascular alterations (mild mitral valve prolapse), cognitive impairment |
| PR008 | DEL1-58:c.(?_-1)_(*1_?)del (p.0?) | 2.0 |  | none |
| PR009 | DEL1-58:c.(?_-1)_(*1_?)del (p.0?) | 28.0 |  | none |
| PR010 | DEL1-58:c.(?_-1)_(*1_?)del (p.0?) | 24.0 |  | none |
| PR011 | DEL1-58:c.(?_-1)_(*1_?)del (p.0?) | 3.0 |  | none |
| PR012 | DEL1:c.(?_-1)_(60+1_61-1)del (p.0?) | 36.0 |  | Macrocephaly, plexiform neurofibroma, cognitive impairment |
| PR013 | DEL1:c.(?_-1)_(60+1_61-1)del (p.0?) | 13.0 |  | Epilepsy, cognitive impairment |
| PR014 | c.499_502delTGTT(p.Cys167Glnfs*10) | 31.0 |  | Bone dysplasia (femur/tibia) |
| PR015 | c.499_502delTGTT(p.Cys167Glnfs*10) | 4.0 |  | Macrocephaly, symptomatic optic glioma, cardiovascular alterations (cardiac fibromas), cognitive impairment |
| PR016 | c.499_502delTGTT(p.Cys167Glnfs*10) | 28.0 |  | Plexiform neurofibroma, cardiovascular alterations (venous malformation of right cerebellar hemisphere and hydrocephalus) |
| PR017 | c.499_502delTGTT(p.Cys167Glnfs*10) | 17.0 |  | Cardiovascular alterations (hypertension) |
| PR018 | c.499_502delTGTT(p.Cys167Glnfs*10) | 55.0 | Breast cancer | none |
| PR019 | c.499_502delTGTT(p.Cys167Glnfs*10) | 1.0 |  | none |
| PR020 | c.499_502delTGTT(p.Cys167Glnfs*10) | 65.0 |  | none |
| PR021 | c.574C>T(p.Arg192*) | 59.0 | MPNST | Cardiovascular alterations (hypertension), cognitive impairment |
| PR022 | c.574C>T(p.Arg192*) | 0.5 |  | none |
| PR023 | c.574C>T(p.Arg192*) | 2.0 |  | none |
| PR024 | c.574C>T(p.Arg192*) | 31.0 |  | none |
| PR025 | c.574C>T(p.Arg192*) | 10.0 |  | none |
| PR026 | c.889-1G>A (p.?) | 33.0 |  | Short stature, dystrophic scoliosis |
| PR027 | c.889-1G>A (p.?) | 38.0 |  | Symptomatic spinal neurofibroma |
| PR028 | c.889-1G>A (p.?) | 0.7 |  | none |
| PR029 | c.910C>T(p.Arg304*) | 19.0 |  | Dystrophic scoliosis, cognitive impairment |
| PR030 | c.910C>T(p.Arg304*) | 8.0 |  | Macrocephaly, bone dysplasia (sphenoid), cognitive impairment |
| PR031 | c.910C>T(p.Arg304*) | 11.0 |  | none |
| PR032 | c.910C>T(p.Arg304*) | 35.0 |  | none |
| PR033 | c.1019_1020delCT(p.Ser340Cysfs*12) | 59.0 | Breast cancer | Cardiovascular alterations (hypertension) |
| PR034 | c.1019_1020delCT(p.Ser340Cysfs*12) | 10.0 |  | none |
| PR035 | c.1185+1G>A (p.Asn355_Lys395del) | 12.0 |  | none |
| PR036 | c.1185+1G>A (p.Asn355_Lys395del) | 27.0 |  | none |
| PR037 | c.1185+1G>A (p.Asn355_Lys395del) | 15.0 |  | none |
| PR038 | c.1260+1604A>G (p.Asn420_Ser421insLeuThrThr*) | 38.0 |  | Bone dysplasia (femur/tibia) |
| PR039 | c.1260+1604A>G (p.Asn420_Ser421insLeuThrThr*) | 54.0 | GIST | none |
| PR040 | c.1318C>T(p.Arg440*) | 19.0 |  | Bone dysplasia (femur/tibia),plexiform neurofibroma, symptomatic spinal neurofibroma |
| PR041 | c.1318C>T(p.Arg440*) | 4.0 |  | Macrocephaly, epilepsy, cognitive impairment |
| PR042 | c.1318C>T(p.Arg440*) | 8.0 |  | Cognitive impairment |
| PR043 | c.1318C>T(p.Arg440*) | 22.0 |  | Macrocephaly, plexiform neurofibroma, cardiovascular alterations (hypertension), symptomatic spinal neurofibroma, cognitive impairment |
| PR044 | c.1318C>T(p.Arg440*) | 39.5 |  | Cognitive impairment |
| PR045 | c.1318C>T(p.Arg440*) | 61.8 |  | Plexiform neurofibroma, cognitive impairment |
| PR046 | c.1318C>T(p.Arg440*) | 78.0 | GIST (multifocal: pancreas and lung), pheocromocitoma | none |
| PR047 | c.1318C>T(p.Arg440*) | 37.0 |  | none |
| PR048 | c.1381C>T(p.Arg461*) | 55.0 |  | Bone dysplasia (femur/tibia), cardiovascular alterations (hypertension) |
| PR049 | c.1381C>T(p.Arg461*) | 11.0 |  | Epilepsy, cognitive impairment |
| PR050 | c.1381C>T(p.Arg461*) | 33.0 |  | none |
| PR051 | c.1381C>T(p.Arg461*) | 8.0 |  | none |
| PR052 | c.1381C>T(p.Arg461*) | 68.0 | Monoclonal gammopathy, colon cancer, cervical intraepithelial neoplasia (CIN) III, thyroid papillary cancer | none |
| PR053 | c.1466A>G(p.Tyr489Cys) | 31.0 |  | Plexiform neurofibroma |
| PR054 | c.1466A>G(p.Tyr489Cys) | 35.0 |  | Dystrophic scoliosis, plexiform neurofibroma |
| PR055 | c.1466A>G(p.Tyr489Cys) | 12.0 |  | Macrocephaly, plexiform neurofibroma, cognitive impairment |
| PR056 | c.1466A>G(p.Tyr489Cys) | 28.0 |  | Plexiform neurofibroma |
| PR057 | c.1466A>G(p.Tyr489Cys) | 35.0 |  | Cognitive impairment |
| PR058 | c.1466A>G(p.Tyr489Cys) | 6.0 |  | Cognitive impairment |
| PR059 | c.1466A>G(p.Tyr489Cys) | 0.8 |  | Bone dysplasia (sphenoid) |
| PR060 | c.1466A>G(p.Tyr489Cys) | 10.0 |  | none |
| PR061 | c.1466A>G(p.Tyr489Cys) | 5.0 |  | none |
| PR062 | c.1466A>G(p.Tyr489Cys) | 36.0 |  | none |
| PR063 | c.1466A>G(p.Tyr489Cys) | 7.0 |  | none |
| PR064 | c.1541_1542delAG(p.Gln514Argfs*43) | 5.0 |  | Symptomatic optic glioma, cognitive impairment |
| PR065 | c.1541_1542delAG(p.Gln514Argfs*43) | 19.0 |  | Macrocephaly, plexiform neurofibroma |
| PR066 | c.1541_1542delAG(p.Gln514Argfs*43) | 29.7 |  | Dystrophic scoliosis, bone dysplasia (femur/tibia), plexiform neurofibroma, symptomatic spinal neurofibroma |
| PR067 | c.1541_1542delAG(p.Gln514Argfs*43) | 23.0 |  | Plexiform neurofibroma, epilepsy (also diagnosed with MELAS) |
| PR068 | c.1541_1542delAG(p.Gln514Argfs*43) | 2.0 |  | Macrocephaly |
| PR069 | c.1541_1542delAG(p.Gln514Argfs*43) | 5.0 |  | none |
| PR070 | c.1541_1542delAG(p.Gln514Argfs*43) | 60.0 |  | none |
| PR071 | c.1541_1542delAG(p.Gln514Argfs*43) | 58.8 |  | none |
| PR072 | c.1756_1759delACTA(p.Thr586Valfs*18) | 3.0 |  | Symptomatic optic glioma |
| PR073 | c.1756_1759delACTA(p.Thr586Valfs*18) | 7.0 |  | Cognitive impairment |
| PR074 | c.1756_1759delACTA(p.Thr586Valfs*18) | 65.0 |  | Dystrophic scoliosis, cardiovascular alterations (hypertension), cognitive impairment |
| PR075 | c.1756_1759delACTA(p.Thr586Valfs*18) | 47.0 | Meningioma | none |
| PR076 | c.1756_1759delACTA(p.Thr586Valfs*18) | 1.0 |  | none |
| PR077 | c.1756_1759delACTA(p.Thr586Valfs*18) | 33.0 |  | none |
| PR078 | c.1885G>A(p.Gly629Arg) | 35.0 | Duodenal carcinoma, small cell carcinosis of peritoneum, pheocromocitoma | none |
| PR079 | c.1885G>A(p.Gly629Arg) | 9.0 |  | none |
| PR080 | c.2033dupC(p.Ile679Aspfs*21) | 58.0 |  | Symptomatic spinal neurofibroma |
| PR081 | c.2033dupC(p.Ile679Aspfs*21) | 33.0 |  | Bone dysplasia(femur/tibia), plexiform neurofibroma |
| PR082 | c.2033dupC(p.Ile679Aspfs*21) | 35.0 |  | Short stature, macrocephaly, plexiform neurofibroma |
| PR083 | c.2033dupC(p.Ile679Aspfs*21) | 12.0 |  | none |
| PR084 | c.2033dupC(p.Ile679Aspfs*21) | 47.0 |  | none |
| PR085 | c.2446C>T(p.Arg816*) | 69.0 | MPNST, carcinoid of the Vater's ampulla | none |
| PR086 | c.2446C>T(p.Arg816*) | 10.0 |  | Macrocephaly |
| PR087 | c.2970_2972delAAT(p.Met992del) | 2.0 |  | none |
| PR088 | c.2970_2972delAAT(p.Met992del) | 35.0 | Breast cancer | none |
| PR089 | c.3457_3460delCTCA(p.Leu1153Metfs*4) | 2.0 |  | Plexiform neurofibroma |
| PR090 | c.3457_3460delCTCA(p.Leu1153Metfs*4) | 50.0 | pheocromocitoma | Dystrophic scoliosis |
| PR091 | c.3457_3460delCTCA(p.Leu1153Metfs*4) | 27.0 |  | Bone dysplasia (sphenoid), plexiform neurofibroma |
| PR092 | c.3457_3460delCTCA(p.Leu1153Metfs*4) | 68.0 |  | Cognitive impairment |
| PR093 | c.3457_3460delCTCA(p.Leu1153Metfs*4) | 70.0 | GIST (duodenum) , rectal adenocarcinoma | none |
| PR094 | c.3457_3460delCTCA(p.Leu1153Metfs*4) | 5.0 | Neuroblastoma (uterus-ovary) | none |
| PR095 | c.3665delC(p.Pro1222Leufs*2) | 6.0 |  | none |
| PR096 | c.3665delC(p.Pro1222Leufs*2) | 10.0 | Juvenile myelomonocytic leukemia | Macrocephaly |
| PR097 | c.3721C>T(p.Arg1241*) | 2.0 |  | Cognitive impairment |
| PR098 | c.3721C>T(p.Arg1241*) | 25.0 |  | Cardiovascular alterations (hypertenion) |
| PR099 | c.3826C>T(p.Arg1276*) | 19.0 |  | Bone dysplasia (femur/tibia), plexiform neurofibroma, cognitive impairment |
| PR100 | c.3826C>T(p.Arg1276*) | 55.0 |  | Cardiovascular alterations (vertebral aneurysma) |
| PR101 | c.3826C>T(p.Arg1276*) | 10.0 |  | Cognitive impairment |
| PR102 | c.3826C>T(p.Arg1276*) | 23.0 | Pilomyxoid astrocytomas | none |
| PR103 | c.3826C>T(p.Arg1276*) | 1.0 |  | none |
| PR104 | c.3826C>T(p.Arg1276*) | 55.0 | Malignant glioma | none |
| PR105 | c.4084C>T(p.Arg1362*) | 77.0 |  | Cognitive impairment |
| PR106 | c.4084C>T(p.Arg1362*) | 21.0 |  | Bone dysplasia (femur/tibia), plexiform neurofibroma |
| PR107 | c.4267A>G(p.Lys1423Glu) | 2.0 |  | Short stature, cardiovascular alterations (congenital heart disease without haemodynamic changes) |
| PR108 | c.4267A>G(p.Lys1423Glu) | 37.0 |  | none |
| PR109 | c.4267A>G(p.Lys1423Glu) | 3.0 |  | none |
| PR110 | c.4267A>G(p.Lys1423Glu) | 1.0 |  | none |
| PR111 | c.4537C>T(p.Arg1513*) | 41.0 |  | Short statur,e plexiform neurofibroma, cardiovascular alterations (hypertension) |
| PR112 | c.4537C>T(p.Arg1513*) | 35.0 |  | Bone dysplasia (sphenoid), plexiform neurofibroma |
| PR113 | c.4537C>T(p.Arg1513*) | 25.0 |  | Macrocephaly, symptomatic optic glioma, symptomatic spinal neurofibroma |
| PR114 | c.4537C>T(p.Arg1513*) | 0.7 |  | Cardiovascular alterations (cardiac malformation ) |
| PR115 | c.4537C>T(p.Arg1513*) | 13.0 |  | Short stature, cognitive impairment |
| PR116 | c.4537C>T(p.Arg1513*) | 27.0 |  | Bone dysplasia (tibia/femur) |
| PR117 | c.4537C>T(p.Arg1513*) | 65.0 | GIST | none |
| PR118 | c.4537C>T(p.Arg1513*) | 0.9 |  | none |
| PR119 | c.5839C>T(p.Arg1947*) | 63.0 |  | Symptomatic spinal neurofibroma |
| PR120 | c.5839C>T(p.Arg1947*) | 31.0 |  | Plexiform neurofibroma |
| PR121 | c.5839C>T(p.Arg1947*) | 30.0 |  | Cardiovascular alterations (hypertension) |
| PR122 | c.5839C>T(p.Arg1947*) | 9.0 |  | Cognitive impairment |
| PR123 | c.5839C>T(p.Arg1947*) | 12.0 | Hypothalamic astrocytoma | Cognitive impairment |
| PR124 | c.5839C>T(p.Arg1947*) | 37.0 |  | Bone dysplasia, cardiovascular alterations (hypertension) |
| PR125 | c.5839C>T(p.Arg1947*) | 41.0 |  | none |
| PR126 | c.5839C>T(p.Arg1947*) | 1.0 |  | none |
| PR127 | c.5839C>T(p.Arg1947*) | 2.0 |  | none |
| PR128 | c.5839C>T(p.Arg1947*) | 4.0 |  | none |
| PR129 | c.5839C>T(p.Arg1947*) | 40.0 |  | none |
| PR130 | c.5839C>T(p.Arg1947*) | 19.0 | Rhabdomyosarcoma | none |
| PR131 | c.5839C>T(p.Arg1947*) | 2.0 |  | Macrocephaly, fetal pyelectasis |
| PR132 | c.5839C>T(p.Arg1947*) | 2.0 |  | none |
| PR133 | c.5844_5845delAA(p.Arg1949Serfs*6) | 48.0 | Meningioma (transitional meningioma, grade I) | Plexiform neurofibroma, epilepsy |
| PR134 | c.5844_5845delAA(p.Arg1949Serfs*6) | 75.5 | lung cancer | Plexiform neurofibroma |
| PR135 | c.5844_5845delAA(p.Arg1949Serfs*6) | 50.0 |  | none |
| PR136 | c.5844_5845delAA(p.Arg1949Serfs*6) | 37.0 | Pylocitic astrocytoma of the cervical spine; sacral ganglioneuroma | none |
| PR137 | c.6579+1G>T(p.Glu2193_Ala2194insVal*) | 21.0 |  | Short stature, dystrophic scoliosis, cognitive impairment |
| PR138 | c.6579+1G>T(p.Glu2193_Ala2194insVal*) | 17.0 |  | Cognitive impairment |
| PR139 | c.6579+1G>T(p.Glu2193_Ala2194insVal*) | 6.0 |  | none |
| PR140 | c.6579+1G>T(p.Glu2193_Ala2194insVal*) | 49.0 |  | none |
| PR141 | c.6579+1G>T(p.Glu2193_Ala2194insVal*) | 1.0 |  | none |
| PR142 | c.6579+1G>T(p.Glu2193_Ala2194insVal*) | 50.0 |  | none |
| PR143 | c.6709C>T(p.Arg2237*) | 51.0 |  | Plexiform neurofibroma, cardiovascular alterations (hypertension) |
| PR144 | c.6709C>T(p.Arg2237*) | 20.0 |  | none |
| PR145 | c.6789_6792delTTAC(p.Tyr2264Thrfs*5) | 41.0 |  | Plexiform neurofibroma, symptomatic spinal neurofibroma |
| PR146 | c.6789_6792delTTAC(p.Tyr2264Thrfs*5) | 33.0 |  | Bone dysplasia (tibia/femur),plexiform neurofibroma |
| PR147 | c.6789_6792delTTAC(p.Tyr2264Thrfs*5) | 30.0 | Sarcoma | none |
| PR148 | c.6789_6792delTTAC(p.Tyr2264Thrfs*5) | 71.0 |  | none |
| PR149 | c.6792C>A(p.Tyr2264*) | 53.0 | IgG-K multiple myeloma | Plexiform neurofibroma, cardiovascular alterations (hypertension and diastolic dysfunction ) |
| PR150 | c.6792C>A(p.Tyr2264*) | 14.0 |  | none |
| PR151 | c.6792C>A(p.Tyr2264*) | 23.0 |  | none |
| PR152 | c.6792C>A(p.Tyr2264*) | 41.0 |  | none |
| PR153 | c.7096_7101delAACTTT(p.Asn2366_Phe2367del) | 7.0 |  | none |
| PR154 | c.7096_7101delAACTTT(p.Asn2366_Phe2367del) | 42.0 | MPNST,breast cancer, rectal and endometrial - patient also diagnosed with Lynch syndrome | none |
| PR155 | c.7285C>Tp.(Arg2429*) | 4.0 |  | Cardiovascular alterations, cognitive impairment |
| PR156 | c.7285C>Tp.(Arg2429*) | 3.0 |  | Short stature, cognitive impairment |
| PR157 | c.7285C>Tp.(Arg2429*) | 1.0 |  | none |
| PR158 | c.7285C>Tp.(Arg2429*) | 30.0 |  | none |
| PR159 | c.7285C>Tp.(Arg2429*) | 37.0 |  | none |
| PR160 | c.7846C>Tp.(Arg2616*) | 56.0 | Myxofibrosarcoma | none |
| PR161 | c.7846C>Tp.(Arg2616*) | 10.0 |  | none |
| PR162 | c.7846C>Tp.(Arg2616*) | 39.0 |  | none |
